# Lost Opportunities

**Published:** 1883-10

**Authors:** 


### HOMŒOPATHIC PHYSICIAN.

A MONTHLY JOURNAL OF MEDICAL SCIENCE.

If our school ever gives up the strict inductive method of Hahnemann, we are lost, and deserve only to be mentioned as a caricature, in the history of medicine."—CONSTANTINE HERING.

Vol. III. OCTOBER, 1883. No. 10.

### EDITORIAL.

LOST OPPORTUNITIES:—In 1844, about forty homœopathic practitioners met in New York city to found a society for "the reformation and augmentation of the materia medica." Why, what need of a society for this purpose? Doubtless these earnest, diligent, and *true* homœopathists reasoned somewhat as follows:

The tens of thousands of allopathists in America and in Europe are engaged in studying anatomy and histology, chemistry and physiology, surgery and pathology, but are almost totally neglecting their therapeutics. Not only do they appear to us to be neglecting their materia medica, but they are totally ignoring and entirely repudiating the homœopathic system and its materia medica. Let us, therefore, said they, organize ourselves into a society for the special purpose of *reforming* and *augmenting* our materia medica. All we desire or need to know of anatomy, chemistry, pathology, diagnosis, surgery, etc., we can learn from the thousands who make these branches their special study. Let us not waste our time in following their footsteps by studying these branches; on the contrary, let us strike out in new paths and make ourselves proficient in our specialty—true therapeutics. Such were, we think, the views of the founders of the once honored American Institute. And during the first years of its existence much good work was done in this, its original line of work. But in late years, as the hard-working veterans died or became incapacitated for further labor, the Institute fell into the hands of a new set; younger—and, as they fondly think, abler (!)—men controlled its work. These gentlemen preferred to desert the study of Homœopathy for that of allopathy—desiring to follow the multitude. To learn how completely the Institute has abandoned the work for which it was organized, one need only look over a volume of its reports. If the journals be correct in their account of the meeting held last June, there were reports from some twelve or fourteen bureaus, comprising about seventy papers. Of these seventy only *three* were on the *treatment* of disease! And only *one* of these homœopathic!

In thus entirely abandoning the work for which it was organized, the Institute throws away a grand opportunity. And they tell us the Institute is prospering! In what way? Why, its numbers are yearly increasing, its transactions are larger and fuller (of allopathy), and its junketings are becoming more and more agreeable. Granted. But what of its work for Homœopathy?

Not satisfied with neglecting the great work of studying Homœopathy, the clique of bosses who now control the Institute, last year determined to boldly declare for eclecticism. So the following eclectic resolution was formulated and put through, as ordered by the bosses:

"That it is the sense of the American Institute that no physician can properly sustain the responsibilities or fulfill all the duties of his professional relations unless he enjoys absolute freedom of medical opinion and unrestricted liberty of professional action, as provided in the code of ethics of this Institute."

The above resolution was passed at the tail end of a session, as before remarked. A goodly number of members of the Institute *since* that meeting have loudly expressed their disapprobation of it, and have declared that it would not have passed a full session, etc., etc. These gentlemen, most of them members of the Institute, were during the past year very bold in their denunciations of the resolution and its object.

Judging them by their words, we expected a determined effort for the rescinding of this obnoxious resolution at the first opportunity. A whole year was given them for preparing for the struggle. The Niagara meeting has come and gone, yet the eclectic resolution still stands on the records of the American Institute unimpeached!

Notice how much more courage the allopaths show. Those of the New York physicians who opposed the new code organized during the year and made a determined effort for its repeal at the next meeting. They failed to rescind the new code resolution, but they, at least, had the courage to fight for what they believed to be right. On the other hand, the true homœopathists of the American Institute loudly decried this infamous resolution *between meetings*, but in sessions they were dumb. So far as the records of the Institute show, there has been no opposition to this resolution, save from *one* brave man! So by their silence they have consented to and ratified the eclectic declaration!!

Thus was a second opportunity lost. Is Homœopathy always to suffer from the apathy of its friends and the treason of its followers?

